# Spatiotemporal Expression of Anticoagulation Factor *Antistasin* in Freshwater Leeches

**DOI:** 10.3390/ijms20163994

**Published:** 2019-08-16

**Authors:** Hee-Jin Kwak, Jeong-Su Park, Brenda Irene Medina Jiménez, Soon Cheol Park, Sung-Jin Cho

**Affiliations:** 1 School of Biological Sciences, College of Natural Sciences, Chungbuk National University, Cheongju, Chungbuk 28644, Korea; 2 School of Life Sciences, Gwangju Institute of Science and Technology (GIST), Gwangju 61005, Korea; 3 Department of Earth Sciences, Paleobiology, Uppsala University, Villavägen 16, 75236 Uppsala, Sweden; 4 Department of Life Sciences, Chung-Ang University, Seoul 156-756, Korea

**Keywords:** *Antistasin*, *Helobdella*, in situ hybridization, expression, hedgehog signaling

## Abstract

*Antistasin*, which was originally discovered in the salivary glands of the Mexican leech *Haementeria officinalis*, was newly isolated from *Helobdella austinensis*. To confirm the temporal expression of *antistasin* during embryogenesis, we carried out semi-quantitative RT-PCR. *Hau-antistasin1* was uniquely expressed at stage 4 of the cleavage and was strongly expressed in the late stages of organogenesis, as were other *antistasin* members. In order to confirm the spatial expression of *antistasin*, we performed fluorescence in situ hybridization in the late stages of organogenesis. The expression of each *antistasin* in the proboscis showed a similar pattern and varied in expression in the body. In addition, the spatial expression of *antistasin* orthologs in different leeches showed the possibility of different function across leech species. *Hau-antistasin1* was expressed in the same region as *hedgehog*, which is a known mediator of signal transduction pathway. Hau-antistasin1 is probably a downstream target of Hedgehog signaling, involved in segment polarity signal pathway.

## 1. Introduction

Hematophagous animals have evolved several mechanisms to counteract the blood coagulation of prey. Injury activates various coagulation factors such as thrombin and factor X to induce blood coagulation and generate blood clots to activate defense mechanisms, such as wound healing. However, animals that ingest blood inject anticoagulants from the salivary glands to inhibit the blood-clotting mechanism, thereby inhibiting several coagulation factors and facilitating sustained bleeding and feeding behavior [1,2,3]. Among the anticoagulants in many animals, antistasin is a serine protease inhibitor (SPI), first discovered in the Mexican leech *Haementeria officinalis* [4,5,6]. This inhibitor, with six conserved cysteine residues and two glycine residues, inhibits the conversion of prothrombin to thrombin by blocking the coagulation factor Xa. Suppression of thrombin cannot regulate platelet activation and the fibrin cascade, and leads to inhibition of thrombogenesis [3,7]. Antistasin plays a multifunctional role, as well as exhibits anti-clotting activity in various animals. Antistasin acts as an anticoagulant in abalone but it is known to exert immune responses to various bacteria. In hydra, which is an ancestral metazoan, antistasin is not only expressed in the digestive tract, but also in the differentiated head [7,8,9]. Antistasin is still restricted to its function as an anticoagulant and studies of animals that do not ingest blood carry insufficient data. As a representative animal, *Helobdella austinensis*, a non-blood-feeding leech, belongs to the family Glossiphoniidae, which consumes hemolymphal fluid (liquidosomatophagous) by inserting a proboscis into the body walls of prey, such as snails and insect larva [2,10]. The presence of antistasin has been identified based on genome information of the sibling species *Helobdella robusta*, but its functional role in embryonic development has yet to be elucidated. We focused on the leech embryo to determine the spatiotemporal transcription of *antistasin*. The possible functional similarity and diversity in leeches was suggested via expression of orthologs in *Alboglossiphonia lata*. We also found that *antistasin* is regulated by Hedgehog signaling, which is a known segment polarity factor.

## 2. Results and Discussion

### 2.1. Phylogeny of Antistasin Orthologs in Serine Protease Inhibitor Groups

**Figure 1:**
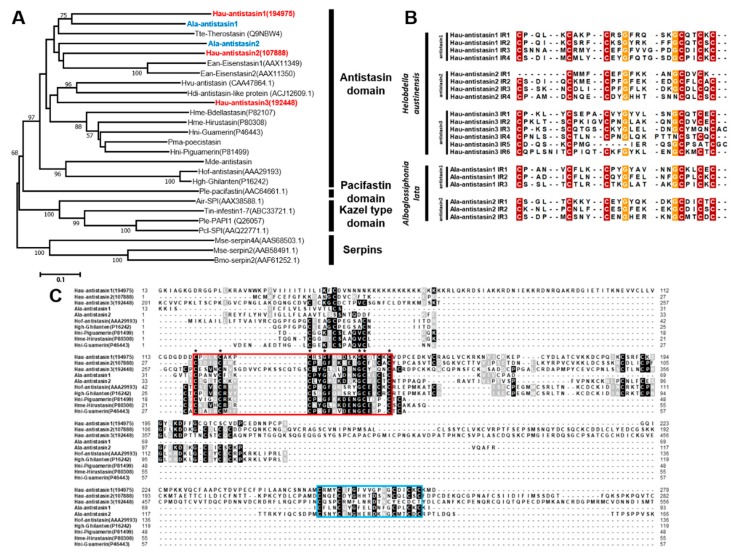
(**A**) Phylogenetic analysis of serine protease inhibitors. *Helobdella* and *Alboglossiphonia* antistasin were clustered with the antistasin family, and two orthologs (antistasin1 and antistasin2) showed homology between two species. Hau-antistasin3, unlike the two genes, was clustered with the out-group in the antistasin family. Hau-antistasin and Ala-antistasin are highlighted in red and blue, respectively. Node values represent percent of bootstrap confidence derived from 1,000 replicates. (**B**) Similarity of internal repeats in antistasin. The antistasin domains contain conserved 5-cysteine and 2-glycine or 6-cysteine and 2-glycine residues. Exceptionally, Hau-antistasin3 carries six internal repeat domains than other types of antistasin. (**C**) Deduced amino acid sequences of *Helobdella austinensis* and *Alboglossiphonia lata* antistasin transcripts with representative antistasins. The red box indicates the conserved sequences of the antistasin family found in other leech species, and the asterisk indicates the cysteine and glycine residues that are consistent within the conserved sequences. The blue box indicates consensus sequences in the antistasin1 and antistasin2 domains of *H. austinensis* and *A. lata*.

We carried out a phylogenetic analysis to determine the relative position of *H. austinensis* and *A. lata* antistasin in serine protease inhibitor (SPI) groups. The neighbor-joining analysis suggested that Hau-antistasin1 and Hau-antistasin2 were related to Ala-antistasin1 and Ala-antistasin2. In contrast, Hau-antistasin3 was grouped with blood-sucking leeches, abalone, and hydra antistasin families (Figure 1A). The phylogenetic analysis showed that Hau-antistasin3 branches out with a common ancestral antistasin group [8,9]. The antistasin1 and antistasin2 orthologs diverged later from antistasin-like inhibitors in *Haementeria* belonging to the same Glossiphoniidae family. Since the genus *Haementeria* is a blood-sucking leech that feeds on mammalian blood [11], our results suggest that the antistasin in the leech originated from blood ingestion and antistasin has been separated by the divergence of species and prey types. In addition, antistasin1 and antistasin2 matched at specific sequences in addition to the conserved antistasin family region, whereas antistasin3 showed no significant match with them (Figure 1C). These characteristic features also suggest that antistasin1 and antistasin2 orthologs may be leech-specific genes that ingest hemolymphal fluid. Similar to other inhibitors belonging to antistasin family, Hau-antistasin1 and Hau-antistasin2 are characterized by four internal repeats. However, Hau-antistasin3 carries six antistasin domains (Figure 1B) and one Kunitz/BPTI domain (data is not shown). These multiple, overlapped inhibitor regions suggest their role as key enzyme inhibitors in *H. austinensis*. In addition, the typical antistasin family is characterized by the presence of conserved 5-cysteine/2-glycine or 6-cysteine/2-glycine residues in the internal repeats of *H. austinensis* and *A. lata* [7] (Figure 1B).

### 2.2. Temporal Expression of Hau-Antistasin Genes during Embryogenesis

**Figure 2:**
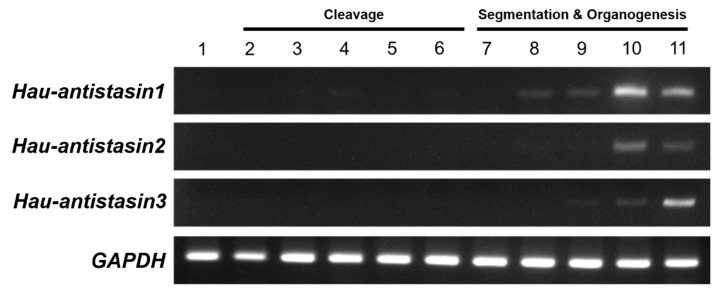
Images of ethidium bromide-stained agarose gels showing replicated developmental semi-quantitative RT-PCR experiments for *Hau-antistasin* genes (30 cycles) and *GAPDH* (35 cycles). Semi-quantitative RT-PCR shows that *Hau-antistasin1*, *Hau-antistasin2*, and *Hau-antistasin3* were highly expressed during organogenesis.

In general, anticoagulants such as antistasin in leeches are known to be secreted from adult salivary glands [12], but the expression pattern at a specific developmental stage has not been elucidated. To determine the temporal expression of *antistasin* transcripts during embryogenesis, we performed semi-quantitative RT-PCR by using *Hau-GAPDH* as an internal control and started with RNA samples derived from all developmental stages to visualize the temporal expression (Figure 2). *Hau-antistasin1*, *Hau-antistasin2*, and *Hau-antistasin3* were expressed at stages 9 to 11 of the organogenesis stages, whereas *Hau-antstasin1* and *Hau-antistasin2* were expressed mainly at stage 8, during germinal band formation. However, *Hau-antistasin1* was weakly expressed at stage 4, during the transition from maternal to zygotic gene [13,14], and at stage 6, before segmentation [15]. These results suggest that *Hau-antistasin1* may play a role in embryonic development during the transition in the cleavage stage of the early embryo. Thus, *antistasin1, anstistasin2,* and *antisatsin3* may play a major role in organogenesis.

### 2.3. Spatial Expression of Hau-Antistasin during Organogenesis

**Figure 3:**
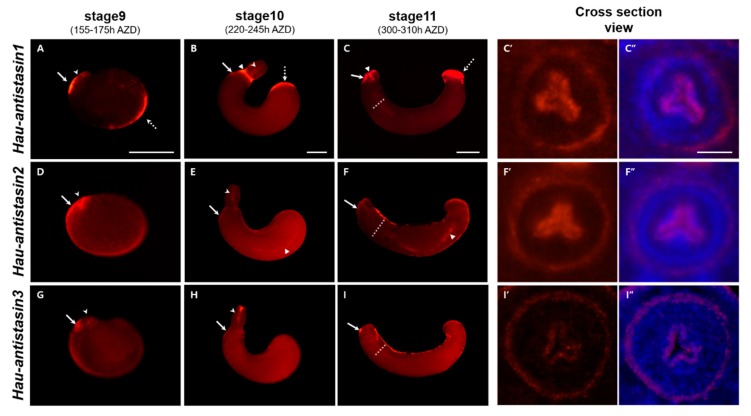
Spatial expression of *Hau-antistasin*. In all panels, the anterior is to the left, dorsal is to the top. (**A**–**C**) Lateral view of *Hau-antistasin1* expressed embryo. At stage 9, *Hau-antistasin1* is expressed in prostomium (white arrowhead), adhesion epithelium to maternal venter (white arrow), and posterior germinal plate (white dot arrow). At stage 10, *Hau-antistasin1* is expressed in the external proboscis, adhesion epithelium (white arrow), proboscis cavity (white arrowhead), boundary of eversion furrow (white triangle), and posterior body (white dot arrow). At stage 11, *Hau-antistasin1* is expressed in the proboscis chamber (white triangle), anterior sucker epithelium (white arrow), and posterior sucker precursor (white dot arrow). (**C’**,**C’’**) To confirm the exact expression region in the proboscis, we dissected the proboscis at stage 11 (white dot line in C). *Hau-antistasin1* is expressed in the outer layer of proboscis and proboscis cavity. (**D**–**F**) At stage 9, *Hau-antistasin2* is expressed in the prostomium (white arrowhead), adhesion epithelium to maternal venter (white arrow), visceral muscle precursor and whole germinal plate. At stage 10, *Hau-antistasin2* is expressed in the proboscis cavity (white arrowhead), adhesion epithelium (white arrow), and visceral muscle (white triangle). At stage 11, *Hau-antistasin2* persisted in the anterior sucker epithelium (white arrow), proboscis cavity, external layer of the proboscis, and gut boundary (white triangle). (**G**–**I**) *Hau-antistasin3* is expressed in the adhesion region (white arrow) and prostomium (white arrowhead) at stage9. At stage 10, *Hau-antistasin3* is expressed in the proboscis cavity (white arrowhead), and adhesion epithelium (white arrow). At stage 11, *Hau-antistasin3* is expressed in the anterior sucker epithelium (white arrow). (**F’**,**F’’**,**I**’,**I’’**) Cross sections of *Hau-antistasin2* and *Hau-antistasin3* show expression similar to *Hau-antistasin1*. Scale bar: A–C = 200 μm; C” = 20 μm.

As in other animals, the presence of antistasin in the leech salivary glands was confirmed only in genome, transcriptome, and proteome analyses of adult specimens [16,17,18], and no spatial expression during embryogenesis has been reported in *H. austinensis*. To confirm the spatial expression based on temporal expression, we visualized *antistasin* transcripts using fluorescent in situ hybridization techniques (Figure 3). In the late stage of organogenesis, three *antistasin* transcripts showed overlapping expression in oral precursor and proboscis. The *Hau-antisatsin1*, *antistasin2*, and *antistasin3* transcripts were strongly expressed in the epithelium attached to the parent venter and boundary of prostomium at stage 9. The overlapped expression persisted in the protruded proboscis epithelium and proboscis cavity at stage 10. After the invagination of the proboscis, the expression continued in the external epithelium of the invaginated proboscis and proboscis cavity. Interestingly, *Hau-antistasin1* showed differential expression at stage 9. Strong expression was observed in the posterior surface of the germinal plate in the embryo and the expression of the posterior body strongly persisted in stage 10. Thereafter, at stage 11, a strong and sustained expression occurred in the posterior sucker precursor. Therefore, *Hau-antistasin1* appears to affect not only the development of the proboscis but also the development of posterior suckers. In the case of *Hau-antistasin2*, the overall expression pattern of the germinal plate was shown at stage 9, indicating expression in the visceral muscle precursor. Subsequently, the intestine was formed, and *Hau-antistasin2* was expressed in the visceral muscle surrounding the yolk; the expression continued at the boundary of the midgut and hindgut at stage 11. *Hau-antistasin2* is involved in gut boundary and muscle tissue development during embryonic development. *Hau-antistasin3* was expressed in the anterior to midbody region of the germinal plate at stage 9, with no characteristic expression except in the proboscis. Thus, *Hau-antistasin3* was associated only with the development of the proboscis.

### 2.4. Co-Linearity and Diversity of Antistasin Orthologs in Glossiphoniidae Leeches

**Figure 4:**
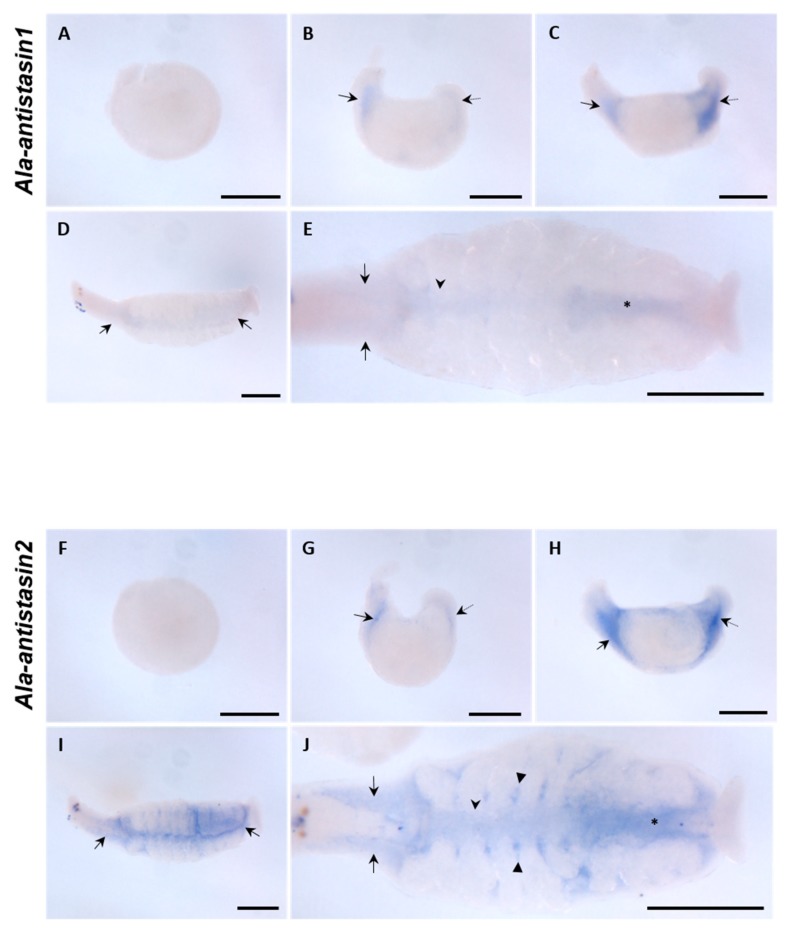
Spatial expression pattern of *Ala-antistasin* transcripts in *Alboglossiphonia lata* embryos. (**A**–**C**,**F**–**H**) *Ala-antistasin1* and *Ala-antistasin2* are expressed in the same region until stage 11. At stage 9, the two *antistasin* transcripts are not expressed. At stage 10, *antistasin* transcripts are expressed in the predicted salivary gland region (black arrow) and hindgut (black dot arrow). During middle stage 11, the same expression appears stronger at the same location. **D**,**E**) At late stage 11, *Ala-antistasin1* is expressed in the salivary gland region (black arrow), midgut (black arrowhead), and hindgut (black asterisk). (**I**,**J**) *Ala-antistasin2* is expressed intensely in the salivary gland (black arrow), midgut (black arrowhead), cecae boundary (black triangle), and hindgut (black asterisk). Scale bar in all images 200 μm.

Phylogenetic analysis and spatiotemporal expression patterns suggest a diverse function of antistasin due to species differentiation. Antistasin belonging to the same family Glossiphoniidae differs according to the food ingested and varying function of the duplicated genes in individual species. We identified the patterns of spatial expression for *antistasin* orthologs in *A. lata* belonging to Glossiphoniidae to complement the above hypothesis. *Ala-antistasin1* and *Ala-antistasin2* showed similar expression patterns during embryogenesis. At stage 9, *Ala-antistasin1* and *Ala-antistasin2* did not show specific expression, unlike *H. austinensis*. However, after proboscis eversion, the orthologs were expressed in the primordial salivary gland region and the hindgut boundary. Even after the proboscis invaginated, the expression continued in the same region, but the expression of *Ala-antistasin1* and *Ala-antistasin2* differed in the late stage 11. *Ala-antistasin1* was expressed weakly in the developing salivary gland, midgut, and hindgut in the late stage 11, whereas *Ala-antistasin2* was strongly expressed in the developing gastric cecae as well as in the salivary gland, midgut, and hindgut (Figure 4). These results indicate that *A. lata antistasin* orthologs during the late organogenesis affect the development of the same organs, and the expression of *Ala-antistasin1* decreases during late embryonic development, leading to organ development at a particular stage. The expression of *Ala-antistasin2* is important for the differentiation of salivary glands and gut in late embryos. In addition, *Hau-antistasin2* and *Ala-antistasin2* were involved in gut differentiation, whereas *Ala-antistasin1* differed from *Hau-antistasin1* in expression site and timing (Figure 3 and Figure 4). The genetic similarity of *antistasin2* between *A. lata* and *H. austinensis* suggests that the function of *antistasin* in the liquidosomatophagous leeches belonging to Glossiphoniidae is partially preserved during gut development. The difference in the expression of *antistasin1* indicated that the gene function can diverge, even though they are homologous genes. These features suggest a partial co-linearity in the physiology of *antistasin* orthologs in the leech, such as gut development, but its role may differ due to species diversification.

### 2.5. Hau-Antistasin1 is Regulated by Hedgehog (hh) Signaling

**Figure 5:**
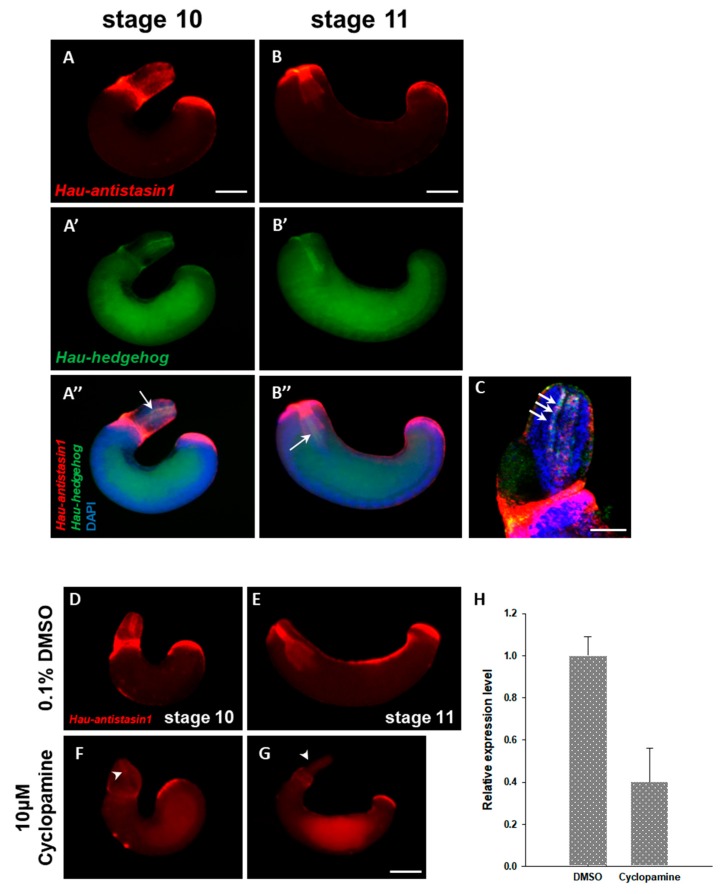
Co-localization of *Hau-hedgehog (hh)* and *Hau-antistasin1*, and cyclopamine treatment test. (**A**,**A’**,**A’’**,**B**,**B’**,**B’’**) *Hau-antistasin1* is expressed in the proboscis cavity during organogenesis. To confirm the overlapping expression region with *hh*, we carried out the double fluorescent in situ hybridization (DFISH). In the proboscis cavity, *hh* expression overlaps with *Hau-antistasin1*. (**C**) High magnification view of overlapped expression pattern. *Hau-antistasin1* and *Hau-hh* are co-localized in the proboscis cavity. (**D**–**G**) Treatment with 10 μM cyclopamine leads to proboscis malformation and failure of proboscis invagination when compared with 0.1% DMSO. *Hau-antistasin1* expression is decreased in the proboscis cavity (white arrowhead). (**H**) Quantitative mRNA expression of *Hau-antistasin1*. Result is shown as mean ± standard deviation. Scale bar: A–B’’, D–G = 200 μm; C = 50 μm.

The various members of the diverse antistasin family show overlapping expression in the proboscis, which is a feeding organ of the leech. It is formed by the dynamic organization of the micromere originating from the mesodermal DM cell to form the complex muscle structure in the proboscis [19]. During formation, the proboscis develops under the control of various transcription factors and signal pathways such as Twist, Hedgehog (hh), and Snail [20,21,22]. Among the numerous signal factors, Hedgehog, known as the segment polarity factor, is associated with organogenesis in the leech and plays a major role in the development of the proboscis [20]. We hypothesized that the *antistasin* genes are regulated by Hedgehog signaling, which plays a major role in the development of proboscis and exhibits a similar pattern of spatial expression as the *antistasin*. *Hau-antistasin1*, which shows an identical temporal expression and strong expression in the everted proboscis, was targeted to co-localize with *hedgehog* during proboscis development. To confirm the expression of the two transcripts during the stages of proboscis development, we used double fluorescence in situ hybridization (DFISH) for co-localization. Within the everted proboscis, *Hau-antistasin1* was expressed in the outer epithelium and proboscis cavity (Figure 5A). The *Hau-hedgehog* gene was expressed in the innermost part of the proboscis cavity that contacts the lumen and the expression positions overlapped with *Hau-antstasin1* (Figure 5A’,C). After the proboscis invagination, the co-localized expression continued. These results indirectly provided spatial evidence suggesting that *Hau-antistasin1* could be regulated by Hedgehog signaling. In order to obtain robust evidence underlying the regulation of *Hau-antistasin1* by Hedgehog signaling, we confirmed *antistasin* expression in an embryo treated with cyclopamine, a Hedgehog signaling inhibitor. In the drug-treated embryos, the proboscis is malformed and are not subsequently invaginated. Compared with the control group, the expression of *Hau-antsatsin1* in the proboscis cavity, outer epithelium, and posterior sucker precursors was decreased. The transcription in the embryos treated with the drug compared with the control group was decreased by more than two-fold. The foregoing results and the reduced transcription level indirectly indicated that *Hau-antistasin1* is regulated by Hedgehog signaling.

In conclusion, the present study suggests that *antistasin* orthologs have evolved with leech species depending on various prey types, and their functions are differentiated within species showing similar prey ingestion but with partially homologous functions. In addition to the enzymatic function of repeated antistasin domains, the spatiotemporal expression of *antistasin* transcripts appears to play a role in the development of specific organs during the organogenesis in the embryo. Hedgehog signal inhibitor treatment indirectly suggests that the *Hau-antistasin1* is a downstream target of Hedgehog signaling which is the key signaling pathway in the organ called the proboscis.

## 3. Materials and Methods

### 3.1. Animals 

*Alboglossiphonia lata* and *Helobdella austinensis* were bred in the Laboratory of Cellular and Development Biology (LCDB), Department of Biology, Chungbuk National University, Republic of Korea. All adult specimens were incubated in a bowl containing a working solution. The specimens were cared once a day by changing the solution and scrubbed manually to get rid of any residual waste. They were maintained in a BOD (Biochemical Oxygen Demand) incubator at 22 °C.

### 3.2. Phylogenetic Analysis and Domain Prediction 

To acquire sequences, we used published gene sequences of a serine protease inhibitor [7,9]. The sequences were aligned and trimmed using the biological sequence editor BioEdit (Available online: http://www.mbio.ncsu.edu/BioEdit/bioedit.html (accessed on 8 July 2019)). The tree was constructed using the neighbor-joining method and p-distance model within the MEGA7 program (Pennsylvania state university, State college, PA, USA). All positions with less than 50% site coverage were eliminated. The domains were investigated by motif an analysis program called MyHits (Available online: http://myhits.isb-sib.ch/cgi-bin/motif_scan (accessed on 8 July 2019)) and PROSITE (Available online: https://prosite.expasy.org/ (accessed on 8 July 2019)).

### 3.3. Gene Identification, Gene Cloning, and Probe Synthesis

Candidates of *Helobdella antistasin* homologs were identified by BLASTP searches, based on the previous study method [14]. The amino acid sequence of *H. officinalis* (accession number: AAA29193) antistasin was used to perform a BLASTP search against the filtered protein model set in the *H. robusta* genome database (http://genome.jgi.doe.gov/Helro1/Helro1.home.html) because of the nearly identical similarity of the genomic DNA coding sequences (>97%) between *H. robusta* and *H. austinensis* [23]. Among the antistasin homologs, three expressed sequence tags (ESTs) were selected and used for experiments. Genes isolated from *H. austinensis* are denoted with a “Hau-” prefix [24]. *A. lata* antistasin homologs were searched based on the RNA database [25] and the deduced protein sequences by ExPASy (https://web.expasy.org/translate/) were confirmed for phylogenetic comparison and domain identification. A BLASTX search of the homologs was performed to confirm the antistasin homologs. We isolated the total RNA from *H. austinensis* and *A. lata* embryos at different developmental stages using TRIzol (Ambion, Austin, TX, USA). We selected the mRNA using RNA using Oligo (dT) primer (Promega, Madison, WI, USA), followed by reverse transcription into cDNA (SuperScript II First-Strand Synthesis System for RT-PCR, Invitrogen, Carlsbad, CA, USA). The investigated leech antistasin genes were isolated using gene-specific primers (*Hau-antistasin1* forward: 5′-CCGCTGTTGAAACGTGCT-3′; *Hau-antistasin1* reverse: 5′-TGAGATCCGCTCTGAACTTCG-3′; *Hau-antistasin2* forward: 5′-GCGAGTTTGGCTTCAAGAAGG-3′; *Hau-antistasin2* reverse: 5′-GTCGCATTCCTGGTTGCAATC-3′; *Hau-antistasin3* forward: 5′-GGCCTACGCATACAGCCAAGCCA-3′; *Hau-antistasin3* reverse: 5′-TGGGCAGTTCAGAGTGCAGCTTG-3′; *Ala-antistasin1* forward: 5′-ATGAGTTTTCCATTAATCTGC-3′; *Ala-antistasin1* reverse: 5′-CCATAGTTGTAACTCTATGCT-3′; *Ala-antistasin2* forward: 5′-TTGTTTCTAGCTGCAGTCAC-3′; *Ala-antistasin2* reverse: 5′-TGTGGTGCTTTGGTCTAGAG-3′). The amplified *antistasin* fragments were subcloned into pGEM T vectors (Promega) and sequenced to confirm their identities. Later, the subcloned inserts were amplified using universal SP6 and T7 promoter primers (SP6 primer: 5′-TATTTAGGTGACACTATAG-3′; T7 primer: 5′-TAATACGACTCACTATAGG-3′). The PCR amplified linear templates were used to synthesize in vitro-transcribed antisense riboprobe (MEGAscript Transcription kit, Ambion, Austin, TX, USA). The genes are available under accession protein numbers 194,975 (*Hau-antistasin1*), 107,888 (*Hau-antistasin2*) and 192,448 (*Hau-antistasin3*). The indicative information can be used to access the gene catalog based on the protein id via the JGI annotation pipeline [26,27]. Sequence information can be retrieved from the JGI genome portal. Dioxigenin-labeled RNA probes were synthesized from the cloned fragments.

### 3.4. Developmental Semiquantitative RT-PCR

The PCR reactions were performed under the following cycling conditions: pre-denaturation at 94 °C for 5 min, followed by 30–40 cycles of denaturation at 94 °C for 30 s, elongation at 72 °C for each sequence length related times, and a final elongation step at 72 °C for 5 min. A 10 μL aliquot of each PCR reaction was removed after 25 cycles, while the remaining material underwent five additional cycles of amplification. The *GAPDH* sequence (protein number: 185,520, *Hau-GAPDH* forward: 5′-GAACGAGGATGGCTACAAGAA-3′; *Hau-GAPDH* reverse: 5′-GTGTACGAGTGGATGGTGG-3ʹ) was used as a standard.

### 3.5. Whole-mount In Situ Hybridization and Cross Sectioning

All in situ hybridization (ISH) and fluorescent ISH procedures were performed as previously described [27,28]. We treated the samples with protease from *Streptomyces griseus* (Sigma-Aldrich, Saint Louis, MO, USA) in PBS, and rinsed three times with glycine dissolved in PBS at room temperature for 5 min. The embryos were post-fixed for 40 min in 4% paraformaldehyde, and finally rinsed three times with PBT. The pre-hybridization was performed at 64.7 °C for a single day in the hybridization buffer (50% Formamide, 5x SSC, 1x Denhardt’s Solution, 0.1% CHAPS, 100 mg/mL Heparin, 0.1% Tween20, 100 mg/mL tRNA). The pre-hybridized buffer was replaced with a fresh hybridization buffer containing 2 ng/mL of the corresponding probe (*antistasin*) and embryos were hybridized at 64.7 °C for 2 days. The washed embryos were incubated at room temperature for 2 h in 1% Blocking Reagent dissolved in PBT (1x PBS plus 0.1% Tween20) and incubated at 4 °C for 16 h with 1/1000 Anti-DIG/AP in 1% Blocking Reagent. After incubation, the color reaction was performed using nitro blue tetrazolium chloride/5-bromo-4-chloro-3-indoyl-phosphate (Roche, Basel, Switzerland) by standard procedures. The stained embryos were dehydrated in ethanol, mounted in plastic embedding solution (PolyBed, Roche, Basel, Switzerland), and examined by bright field microscopy on a Nikon SMZ18 stereomicroscope (Nikon, Minato, TYO, Japan). 

For fluorescent in situ hybridization (FISH), we used the NEN Tyramide Signal Amplification (TSA) Plus Kit (PerkinElmer, Waltham, MA, USA). After washing with PBT, we pre-incubated the embryos in maleic acid buffer (100 mM Maleic Acid, 150 mM NaCl, pH 7.5) for 15 min, the embryos were blocked in 1% Blocking Reagent for nucleic acids for 2 h at room temperature, and incubated at 4 °C for 16 h with 1/1000 Anti-DIG/POD in 1% Blocking Reagent. After incubation, the embryos were rinsed twice with TNT buffer (0.1 M Tris-HCl pH 7.5, 0.15 M NaCl, 0.1% Tween20). Subsequent washes with TNT at room temperature were followed by a single treatment with NEN TSA Plus Amplification Solution. The color reaction was initiated by adding a 1:50 dilution of reconstituted Cyanine-3 Tyramide in NEN Amplification Solution. Embryos were imaged on a Nikon SMZ18 stereomicroscope. After FWISH, the embryos were dehydrated in ethanol (70%, 90% and 100% diluted in 1x PBS) and propylene oxide, followed by infiltration with plastic embedding solution (PolyBed, Polysciences, Inc., Warrington, PA, USA). Embryos were cut by hand using a microtome blade (Leica 818, Leica, Wetzlar, HE, Germany) under an Olympus SZ-STS microscope (Olympus, Shinjuku, Tokyo, Japan). The sections were imaged on an LSM 710 confocal microscope (Carl Zeiss, Oberkochen, BW, Germany).

### 3.6. Drug treatments and qPCR

At stage 7, *H. austienesis* embryos were incubated in HTR media (culture media) including the Hedgehog signaling inhibitor cyclopamine (Sigma-Aldrich, Saint Louis, MO, USA) diluted in DMSO. The total cyclopamine concentration was 10 mM in HTR media and DMSO was 0.1% in solution. The qPCR mixture included a 0.5 μL of *Hau-antistasin1* forward primer (5′-GCTCCTGTGTGGATCCGTGC-3′), and 0.5 μL of *Hau-antistasin1* reverse primer (5′-CGCAATACATTCTACACATGGCG-3′) along with 10 μL 2X q-PCR buffer, 4 μL SYBR green, 1 μL selected cDNA and 2 μL ROX. The PCR was conducted at an annealing temperature of 62 °C, based on standard procedures.
